# Experimental Models to Study Endothelial to Mesenchymal Transition in Myocardial Fibrosis and Cardiovascular Diseases

**DOI:** 10.3390/ijms25010382

**Published:** 2023-12-27

**Authors:** Mohammed Mimouni, Anne-Dominique Lajoix, Caroline Desmetz

**Affiliations:** Biocommunication in Cardio-Metabolism (BC2M), University of Montpellier, 34000 Montpellier, France; mohammed.mimouni@etu.umontpellier.fr (M.M.); anne-dominique.lajoix@umontpellier.fr (A.-D.L.)

**Keywords:** fibrosis, cardiovascular disease, endothelial to mesenchymal transition, in vitro models, animal models

## Abstract

Fibrosis is a common feature of cardiovascular diseases and targets multiple organs, such as the heart and vessels. Endothelial to mesenchymal transition is a complex, vital process that occurs during embryonic formation and plays a crucial role in cardiac development. It is also a fundamental process implicated in cardiac fibrosis and repair, but also in other organs. Indeed, in numerous cardiovascular diseases, the endothelial-to-mesenchymal transition has been shown to be involved in the generation of fibroblasts that are able to produce extracellular matrix proteins such as type I collagen. This massive deposition results in tissue stiffening and organ dysfunction. To advance our understanding of this process for the development of new specific diagnostic and therapeutic strategies, it is essential to develop relevant cellular and animal models of this process. In this review, our aim was to gain an in-depth insight into existing in vitro and in vivo models of endothelial to mesenchymal transition in cardiovascular diseases with a focus on cardiac fibrosis. We discuss important parameters impacting endothelial to mesenchymal transition, and we give perspectives for the development of relevant models to decipher the underlying mechanisms and ultimately find new treatments specific to fibrosis happening in cardiovascular diseases.

## 1. Introduction

Cardiovascular diseases (CVD) refer to a range of disorders that specifically impact the circulating system (heart and vessel network). According to the World Health Organization (WHO), CVD is a major cause of death, accounting for an estimated 17.9 million people every year and representing approximately 30% of deaths worldwide (WHO 2023). Myocardial fibrosis represents a dynamic restructuring of the extracellular matrix (ECM) in response to cardiac stress. Two types of mechanisms have been involved in cardiac fibrosis: “replacement” or “reparative” fibrosis is implicated in the reparative mechanisms that unfold during the acute phase of injuries, such as myocardial infarctions, pulmonary arterial hypertension or congenital heart disease, in order to locally replace dying cardiomyocytes [1]. In stark contrast, “reactive” or “diffuse” myocardial fibrosis corresponds to ECM remodeling developing gradually over time in response to chronic pressure overload and results in the progressive deposition of collagen in the interstitial and perivascular regions [1,2,3]. This persistent collagen deposition disrupts the normal function of either the left or the right ventricle of the heart, leading to ventricular stiffness and poor electrical conductance of the myocardium. Left ventricle reactive fibrosis occurs more specifically in chronic pathological conditions, such as systemic hypertension, aortic stenosis, diabetes mellitus [2], obesity [4], and cardiorenal syndrome [5] and is a key contributing factor leading to arrhythmias and progression to heart failure [1]. Therefore, investigation of the cellular and molecular mechanisms involved in the initiation and progression of myocardial fibrosis is of major importance to prevent its onset in high-risk but not yet diseased patients. 

Endothelial to mesenchymal transition (EndMT) is a complex, vital process that occurs during embryonic formation and plays a crucial role in cardiogenesis, specifically in cardiac valve formation. This transdifferentiation process is characterized by the loss of endothelial fate and the acquisition of mesenchymal features, such as fibroblasts secreting extracellular matrix proteins. EndMT has emerged as a fundamental process implicated in cardiac fibrosis [6,7,8,9,10]. EndMT has also been shown to be involved in many cardiovascular diseases, including pulmonary arterial hypertension [7,8,11,12], atherosclerosis [13], diabetes mellitus [14,15,16,17], and chronic kidney disease [18]. However, the precise contribution of EndMT to cardiac fibrosis and CVD remains unclear, and efforts must be made to clarify those mechanisms. It is, therefore, essential to establish relevant cellular and animal models to elucidate mechanisms involved in this phenomenon. Here, we provide an exhaustive review of the literature concerning the existing cellular and animal models of EndMT in cardiac fibrosis and also fibrosis affecting other target organs of CVD. Finally, we present prospects for the development of EndMT models to improve our knowledge of the mechanisms involved in the initiation and progression of fibrosis to be able to establish effective therapeutic strategies. 

## 2. Endothelial to Mesenchymal Transition in Cardiovascular Diseases

**Figure 1:**
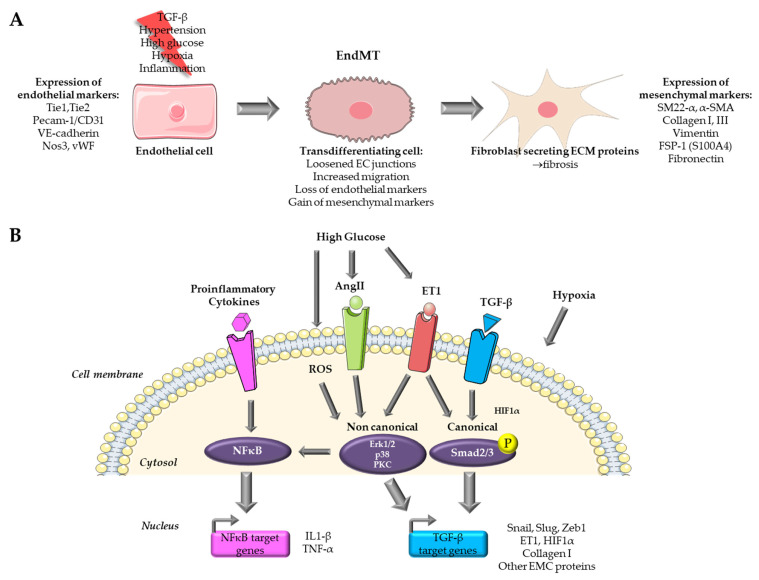
Overview of pathways targeted for the study of EndMT in cardiovascular diseases. (**A**) Endothelial to mesenchymal transition process. (**B**) Pathways targeted in in vitro models for the study of EndMT. Parts of the figure were drawn by using pictures from Servier Medical Art.

Endothelial-mesenchymal transition is a complex process that involves a transition from endothelial cells (ECs) towards a mesenchymal-like phenotype in response to a range of specific stimulators. Cells undergoing EndMT adopt a spindle-shaped morphology that facilitates their unrestricted movement within the ECM because of the loss of their cellular polarity and downregulation of adherent and tight junction proteins. This mobility enables them to assemble the connective tissue crucial for organ function [8]. EndMT mainly occurs during embryonic development, particularly in the early stages of cardiac septum and valve formation [8,9]. It is generally accepted that this transition is characterized by the loss of endothelial markers, such as platelet endothelial cell adhesion molecule (Pecam-1/CD31), vascular endothelial cadherin (VE-cadherin), von Willebrand factor (vWF), and the acquirement of a mesenchymal (fibroblastic-like) phenotype, characterized by the expression of mesenchymal markers including SM22-α (Smooth muscle protein 22-alpha), vimentin, α-SMA (Actin alpha 1 skeletal muscle), type I collagen, FSP-1 (Fibroblast specific protein-1, i.e., S100A4), fibronectin [6,8] (Figure 1A). Nevertheless, when examining EndMT at the molecular level, there is currently no consensus on criteria allowing the precise definition of this process. This lack of standardization poses a growing challenge, as it leads to a lack of uniformity and limited comparability of data issued from various model systems and research publications [9]. 

Several signaling pathways have been documented to initiate and regulate EndMT during both physiological and pathological conditions, including bone morphogenetic protein (BMP)–transforming growth factor (TGF), vascular endothelial growth factor A (VEGFA), epidermal growth factor (EGF), fibroblast growth factor (FGF), Notch, platelet-derived growth factor (PDGF), Wnt/β-catenin, calcineurin–NFAT, transcription factor GATA4-mediated transcriptional regulation, endothelin-1 (ET-1) and inflammatory signaling. These pathways have been extensively studied in the latest reviews [2,6,8,9]. However, the implication of EndMT in fibrosis remains unclear. Through the analysis of in vitro and in vivo EndMT models, this review will attempt to shed some light on the link between EndMT and fibrosis.

## 3. Existing In Vitro Models of Endothelial to Mesenchymal Transition in Cardiovascular Diseases

The majority of in vitro models for EndMT utilize primary ECs. In vitro models offer several advantages over their in vivo counterparts, including ease of accessibility, cost-effectiveness, and the potential for achieving highly reproducible outcomes. Nevertheless, it is important to acknowledge that in vitro models do have limitations when compared to in vivo models, and these limitations may impact the extrapolation of findings derived from such models. An overview of the signaling pathways targeted by in vitro models for the study of EndMT in cardiovascular diseases is presented in Figure 1B.

### 3.1. Cytokine Based In Vitro Models 

#### 3.1.1. TGF-β Signaling

Among the growth factor families, TGF-β emerges as the most significant cytokine-inducing transdifferentiation of EC (recent review [19]). TGF-β plays a pivotal role at the early embryonic stages in guiding the atrioventricular canal formation [20]. TGF-β is secreted by both fibroblasts and macrophages and stimulates collagen and ECM component production by enhancing EndMT, which in turn leads to fibrotic lesions [21]. The initiation of TGF-β-induced EndMT signalization following the canonical pathway is achieved through the binding of TGF-β isoforms to its receptor TGFBR1/2, activating its intrinsic kinase activity. This cascade of kinase/phosphorylation activity continues within the cell to the nucleus, across phosphorylation of downstream effectors, including receptor-activated Smad (R-Smads) and particularly Smad2/3, leading to the formation of a hetero-oligomeric complex with Smad4. This promotes their translocation in the nucleus where the complex binds to target gene promoters, inducing the expression of transcription factors like Snail, Slug, and Twist, which leads to the up-regulation of numerous EndMT-involved genes [8,19]. As for the canonical pathway, emerging evidence highlights the involvement of non-canonical pathways in the signaling of TGF-β-induced EndMT through the activation of a Smad-independent kinase complex, which requires several other downstream effectors, such as the mitogen-activated protein kinase (MAPK), PI3K, and PKC-δ. Indeed, TGF-β has been shown to induce the activation of MAPK cascades in a fibrosis context, triggering extracellular signal-regulated kinase (ERK), c-Jun N-terminal kinase (JNK), and p38 MAPK. These activated downstream effectors can transduce signals to the nucleus, leading to an increased expression of transcription factors, thereby stimulating the up-regulation of EndMT-associated gene expression [19]. While the precise mechanisms governing these pathways exhibit diversity and remain partially understood, the majority of these pathways ultimately intersect by modulating transcription factors, such as Snail and Twist, to inhibit the expression of EC markers and to induce the expression of mesenchymal proteins.

#### 3.1.2. TGF-β Isoform Specific Effects to Induce EndMT

Varying degrees of potency in promoting EndMT has been attributed to the three TGF-β isoforms, namely TGF-β1, TGF-β2, and TGF-β3. Among them, TGF-β2 appears to be the isoform with the greatest potential for inducing EndMT, as highlighted during embryonic heart development [22] and on immortalized human dermal microvascular endothelial cells (HMVEC) [23,24]. Exposure for 72 h to 1 ng/mL TGF-β1 and TGF-β3 led to an increase in the expression of TGF-β2, while TGF-β2 treatment did not. This suggests that EndMT induced by TGF-β1 and TGF-β3 is the result of secondary effects mediated through the secretion of TGF-β2. Moreover, TGF-β2 increases phosphorylation (activation) of Smad2/3 (canonical) and p38 MAPK (non-canonical) at a higher level than TGF-β1 and TGF-β3. Finally, silencing TGF-β2 attenuated the expression of EndMT markers in cells treated with TGF-β1 and TGF-β3. This, therefore, suggests that TGF-β1 and TGF-β3-induced EndMT involves a paracrine loop mediated by TGF-β2 [23]. Interestingly, studies carried out over the last 10 years have used either TGF-β2 [23,24,25,26] or TGF-β1 on endothelial cells from different origins [27,28,29,30,31,32,33,34] and, more rarely, TGF-β3 [23,35].

#### 3.1.3. EndMT Induction Level Depend on Endothelial Cell Origin

Most in vitro models use endothelial cells of different anatomical origins treated with a member of the TGF-β family. In these models, EndMT is characterized by four characteristics: (1) simultaneous expression of endothelial and mesenchymal markers, (2) heightened cellular migratory capabilities, (3) diminished expression of endothelial traits, including reduced leukocyte adhesion and impaired tubule formation, and (4) increased manifestation of mesenchymal/myofibroblastic traits, such as augmented collagen production and enhanced contractility [36]. Several EC models are widely used, such as human umbilical vein ECs (HUVEC), aortic EC (HAEC), coronary EC (coronary artery CAEC, or microvascular CMVEC), cardiac EC (microvascular CMEC, atrial endocardial AEEC), pulmonary artery EC (PAEC), and dermal EC (HMEC). Two studies provide interesting evidence that cells from different anatomical origins do not have the same potential to enter transdifferentiation. Among the origins tested (coronary artery, aortic, umbilical vein, pulmonary artery), cells of aortic and coronary origin showed the best responses to induction by TGF-β2 treatment. This results in an increase in several mesenchymal markers, such as type I collagen, actin alpha cardiac muscle (ACTC), SM22-α, Calponin 1 (CNN1), Snail, and maintenance of endothelial markers such as Pecam-1 and VE-cadherin. This is also accompanied by a morphological and functional change, with a diminished ability to form vessel-like structures [25]. In cells of aortic origin, the induction of EndMT is accompanied by significant activation of the non-canonical pathway via ERK1/2. In aortic and coronary cells, induction is potentiated by Snail overexpression in combination with TGF-β2 treatment, which is less obvious for HUVEC [37] or PAEC [25,37,38]. Moreover, cells of umbilical and pulmonary origin showed a delay or resistance to enter transdifferentiation and do not produce type I collagen (something we also observed in the laboratory, unpublished data). It is, therefore, clear that the origin of EC plays a role in their ability to enter EndMT and that more information is required about these origins, as well as the conditions under which EndMT is induced.

#### 3.1.4. Induction of EndMT by Co-Treatment with Proinflammatory Signals

The second most widely used model is that of EC treated with a combination of TGF-β and a proinflammatory cytokine, like IL1-β, TNF-α, or both. Indeed, the inflammatory context found in many cardiovascular diseases has been shown to promote cardiac fibrosis [3]. Most studies demonstrate that induction of EndMT is possible using TGF-β isoforms in combination with a proinflammatory cytokine [35,38,39]. This is the case for HUVEC, where costimulation with IL-1β and TGF-β1 or 2 caused synergistic induction of EndMT accompanied by overexpression of the transcription factor NF-κB, an increase in its nuclear translocation [40,41], and type I collagen expression, which was also the case in PAEC [32,38]. Cotreatment with TNF-α also induced EndMT [42]. Treatment with IL-1β and TNF-α without TGF-β was also able to induce expression changes related to EndMT in HUVEC [41,43], suggesting that these cells may be more susceptible to inflammatory stimuli. This was also demonstrated in other organ fibrosis disorders, such as intestinal fibrosis [44]. However, in that particular case, no significant change in their migratory capacity was observed, confirming a resistant or delayed capacity of entering EndMT in HUVEC and PAEC [38], showing once again that cell origin plays a role in the ability to enter EndMT. Finally, angiotensin II (Ang II), a major fibrosis inducer [3], is also able to induce EndMT by activating NFκB signaling and pro-inflammatory cytokine production in HUVEC [45,46,47,48] in HMVEC [49] and in HCAEC [50]. AngII also induces and activates the TGF-β axis [3]. In short, proinflammatory signals, whether or not associated with TGF-β, are capable of inducing EndMT in ECs and represent widely used in vitro models.

### 3.2. Hypoxia-Based In Vitro Models

Chronic hypoxia plays a pivotal role in the development of cardiac fibrosis and is induced by a reduced density of microvessels, which subsequently impacts oxygen delivery and increased oxygen consumption due to the activation of inflammatory cells and fibroblasts. Chronic hypoxia independently contributes to abnormal ventricular remodeling and the onset of cardiac fibrosis [51]. Hypoxia has the capability to trigger EndMT via HIF1 signaling, even in the absence of TGF-β, as it directly regulates Snail expression through HIF1α in HCAEC [52]. Several publications, such as MVEC [53], HCAEC [52], and HUVEC [54,55,56], use cells under hypoxia as an EndMT model. Interestingly, hypoxia is widely used on PAEC [57,58,59] and PMVEC of pulmonary origin [60]. Indeed, extensive vascular remodeling occurs in pulmonary arterial hypertension (PAH), where neointimal and medial thickening results from fibrosis in the pulmonary arteries [12]. In vitro models of hypoxia-induced EndMT were developed in the last 10 years and allowed elucidation of signaling cascades activated in response to hypoxia in PAH (reviewed in [12]). Hypoxia conditioning of EC, therefore, appears to be a relevant in vitro model for studying EndMT, especially in PAH.

### 3.3. High Glucose Based In Vitro Models of Organ Fibrosis in Diabetes Mellitus 

One of the consequences of hyperglycemia is vascular dysfunction, which is a main component of diabetes mellitus, leading to both micro- and macro-vascular complications, and which affects several organs such as the kidney (diabetic nephropathy), heart (diabetic cardiomyopathy), retina (diabetic retinopathy), resulting in organ fibrosis. EndMT has been identified as an early important potential trigger in diabetic complications, as ECs are among the first to be damaged by hyperglycemia (recent review in [17]). High glucose activates a variety of EndMT-inducing pathways, such as TGF-β (through the PKC pathway), ET-1, and inflammatory signals. EndMT models were therefore designed with EC from these different origins and treated with high glucose concentrations in order to mimic hyperglycemia. HUVEC is widely used as a model for diabetes-associated cardiac fibrosis [14,61,62,63,64,65,66,67,68,69,70]. In particular, Yu et al. showed that 24 h treatment with 30 mM glucose resulted in the expression of the mesenchymal markers α-SMA and type I collagen in HUVEC. Endothelial markers (Pecam-1, VE-cadherin) were under-expressed more slowly or at higher glucose concentrations, reflecting a delay in the regulation of these markers during glucose-mediated EndMT. Finally, mesenchymal cells were obtained at 60 mM glucose and after 48 h of treatment. High glucose-induced TGF-β1 production from 48 h of treatment [63] shows that high glucose-mediated EndMT involves the TGF-β pathway. In vitro studies using HUVEC are usually combined with in vivo studies on animal models of diabetic disease. Aortic EC and CAEC are also used to study diabetic cardiomyopathy [71,72,73,74,75]. It is worth noting that to study EndMT in diabetic nephropathy and kidney fibrosis, glomerular endothelial cells (GEnC) are specifically used [76,77,78,79,80,81,82,83]. Finally, human retinal EC is used to study diabetic retinopathy [84,85,86,87,88].

Recently, a new 3D model of the aortic valve was developed in order to enhance the comprehension of the dynamic interplay between ECs and their microenvironmental matrix in the context of hyperglycemia [89]. The authors used hydrogel containing extracellular matrix from porcine aortic root and human valve cells. They cultivated valve EC on the hydrogel’s surface and valve interstitial cells within the hydrogel, then exposed this 3D structure to high glucose conditions. The authors observed a reduced expression of endothelial markers Pecam-1 and VE-cadherin and increased expression of mesenchymal markers α-SMA and Vimentin. There was also a loss of intercellular junctions and an enhanced expression of inflammatory molecules. Finally, valvular ECs showed enhanced monocyte adhesion via a mechanism involving adhesion molecules such as ICAM-1 and VCAM-1, showing dysfunctionality. The 3D model contains an ECM mainly composed of collagen I and III, similar to the aortic valve structure [89]. This model could also be interesting for cardiac myocardial fibrosis because ECM has a similar composition.

### 3.4. Important Parameters to Consider

#### 3.4.1. Purity of Cell Preparation

Cells can be sourced from a variety of suppliers, or they can be isolated from animals, in which case the question of the preparation purity arises. Indeed, contaminating cells (smooth muscle cells, fibroblasts, or pericytes), even present in very low quantities, will express mesenchymal markers such as α-SMA, a frequently employed marker for EndMT, leading to false positive results. Moreover, cell origin conditions determine the process intensity, as evoked earlier, with cells of venous origin showing later differentiation and of lower intensity compared to others. Therefore, the origin of the cells must be adapted to their intended use, and purity must be maximized; otherwise, the results will be irrelevant.

#### 3.4.2. Heterogeneity of Induced EndMT Phenotypes

Depending on treatment conditions, variable-induced EndMT phenotypes can be obtained in vitro. Monteiro et al. treated HUVEC with IL-1β (1 ng/mL), TGF-β2 (10 ng/mL) or both during 7 days. TGF-β2 alone was able to induce Slug (SNAI2) expression, but only the cotreatment was able to induce a decrease in Pecam-1 expression and an increase in Collagen I and α-SMA, along with a decreased proliferation. However, as no change in migration capacities was observed, this process was described as early EndMT [38]. Another report treated ECs of artery origin with TGF-β concentrations varying from 2 to 10 ng/mL during 5 days without culture media change and observed an increase in α-SMA but no loss of Pecam-1 expression. Based on these expression data, this reflects an intermediate phenotype of EndMT with cells having partially undergone transition [26]. This partial transdifferentiation phenotype could be involved in cardiac fibrosis, but its importance remains to be evaluated [90]. The cells that achieve complete transdifferentiation might not indeed correspond to the number of cells undergoing EndMT because EndMT is a reversible and dynamic process [91]. In vitro models of these intermediate stages of EndMT need to be developed. Technical issues also impact induced EndMT phenotypes. Indeed, the culture conditions used for EC in the literature are highly heterogeneous. Moreover, some authors underline the lack of consistent information regarding culture conditions, especially serum conditions, with some studies working without serum and some others with 2 to 10% serum. Their personal observations revealed that serum-free conditions induced massive EC death as early as 24 h, a phenomenon that we also observed in our laboratory, which led us and others to use serum-containing medium [26]. 

Finally, the heterogeneity of induced EndMT phenotypes may reflect the lack of functional assays that are absolutely necessary for attesting the transition process. First, expression assays, in addition to endothelial and mesenchymal marker expression data, should show evidence of coexpression of these markers in the same cells along with morphological changes. Second, functional assays should be performed, such as loss of endothelial properties (tubule formation, leukocyte adhesion) and increase in migratory capacity. Therefore, cells that maintain endothelial markers and endothelial functions have experienced partial EndMT. Cells that have lost endothelial markers and functions and have acquired mesenchymal features have undergone complete EndMT [36].

In conclusion, in vitro models are mainly used to decrypt molecular mechanisms involved in EndMT, especially in the context of fibrotic diseases. We still need to better understand the conversion of endothelial cells into mesenchymal cells and the different stages of the process. 

## 4. Existing In Vivo Models of EndoMT in Cardiovascular Diseases

**Figure 2:**
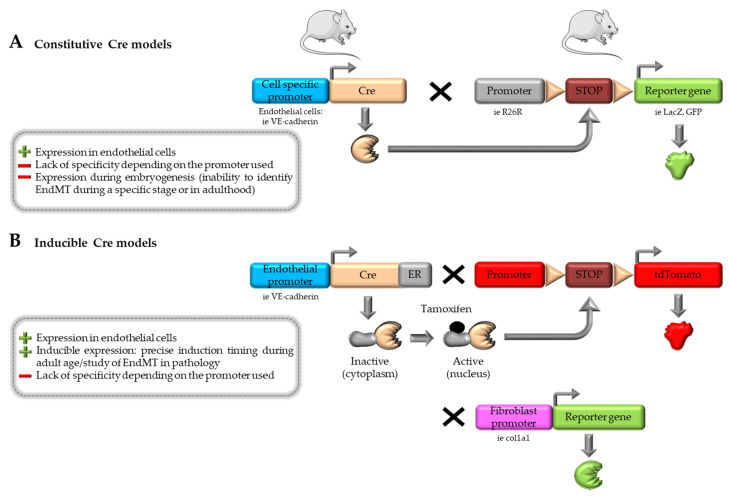

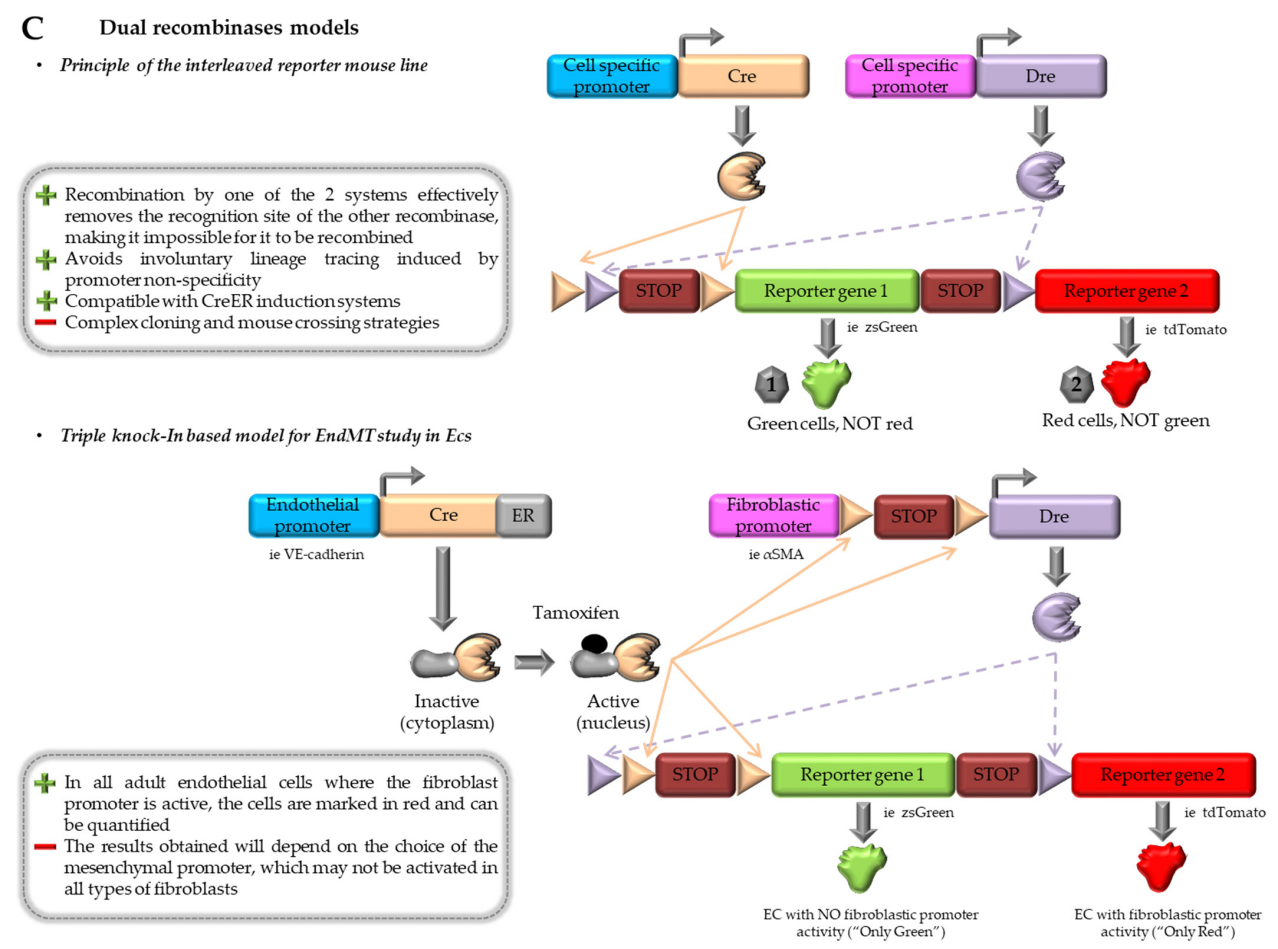
Existing in vivo models for the study of EndMT in cardiovascular diseases. (**A**) Principle for the generation of constitutive Cre animal models. (**B**) Principle for the generation of inducible Cre animal models. (**C**) Principle for the generation of dual recombinase animal models (upper panel) and its application in the study of EndMT (lower panel). Parts of the figure were drawn by using pictures from Servier Medical Art.

Most in vivo studies focus on CVD pathophysiology and use models that mimic the main features of the disease being studied. However, in those animal models, it is rarely possible to follow the fate of EC. The strategies used are, therefore, the same as in vitro, i.e., demonstrating the coexpression of mesenchymal and endothelial markers or changes in cell morphology. With the aim of better characterizing the role of EndMT in cardiac fibrosis and other CVD pathologies, lineage-tracing animal models have been progressively developed and used. These models have provided additional insights into the EndMT process. These models are summarized in Figure 2. 

### 4.1. Cre-loxP System 

To track the lineage origins of endothelial cells in vivo, genetic fate mapping techniques are applied using murine endothelial-specific Cre-lox lineage tracking systems (Figure 2A). These mice typically carry a Cre recombinase under the control of an endothelial-specific promoter. Upon Cre activation, as early as the embryonic stage, a reporter protein is expressed under the control of a promoter, marking all cells of endothelial origin [6]. In a murine acute pressure-overload model (consisting of aortic banding), Zeisberg et al. utilized Tie1Cre;R26RstoplacZ double transgenic mice to track EC undergoing EndMT. The Tie1 gene encodes a tyrosine kinase receptor expressed by EC. In this animal model, lacZ expression occurs in cells of endothelial origin independently of subsequent changes. In order to track EndMT in these lacZ-positive cells, they labeled FSP1 (S100A4) as a marker of fibroblasts by confocal immunofluorescence. Their findings demonstrated that ECs undergo EndMT and contribute to approximately 27–35% of the total cardiac fibroblast population during cardiac fibrosis following aortic banding [92]. Another study used a similar model knocking out the ET-1 gene from EC using the Tie2 promoter in a mouse model of diabetes mellitus. Cardiac fibrosis induced in ET-1-positive diabetic mice was abolished when ET-1 was knocked out. The authors observed that 15 to 20% of fibroblasts coexpressed both CD31 (Pecam-1) and FSP-1 in the hearts of diabetic mice, whereas in ET-1 KO mice, these cells were rarely detected [14]. Although providing interesting insights, the Cre-loxP model lacks relevance for differentiating fibroblasts originating from the embryonic endocardium through EndMT from those derived from EndMT in adulthood and the consequent onset of cardiovascular pathology. It is, therefore, difficult to conclude whether EndMT is involved in pathology in these models. Moreover, this constitutive Tie1 or Tie2-induced Cre expression also labels circulating monocytes and other leukocytes that may be involved in fibrosis. Additionally, FSP1 lacks specificity, as it labels only a subset of fibroblasts and is expressed by other cell types, including endothelial and immune cells [92,93]. Finally, since this genetic labeling is irreversible and heritable, the progeny of the Cre-marked cells continue to express the reporter gene, whether or not they actively express Cre [94].

### 4.2. Inducible Cre-loxP System and Multiple Reporter Models

Inducible Cre models were developed to improve the characterization of EndMT during cardiac fibrosis (Figure 2B). These models allow tissue-specific Cre expression within a defined timeframe when the inducer tamoxifen is administered to mice. Moore Morris et al. generated mice expressing Cre recombinase fused to the estrogen receptor (ER) under the control of the VE-cadherin promoter (endothelial) in mice that express tdTomato fluorescence (RosatdT) following Cre-mediated recombination. When tamoxifen is administered to mice, Cre is expressed, allowing the Stop codon to be removed in the tdT gene, therefore labeling EC with the Tomato gene. Mice were further crossed with col1a1-GFP mice (Figure 2B). Thus, after tamoxifen induction, the presence of double-positive cells can reveal a transition state during EndMT. The results of this study showed that EndMT occurred during embryonic stages to give rise to cardiac fibroblasts. However, adult endothelium did not give rise to cardiac fibroblasts during pressure overload-induced fibrosis [95]. Also, this study (along with others) identified type I collagen (Col1a1) along with PDGFRα (platelet-derived growth factor receptor-α) as being relevant markers for fibroblasts in the heart [96]. 

In order to identify the fibroblast origin after myocardial infarction, Kanicicak and collaborators performed a lineage tracing study using inducible Cre under the control of transcription factor 21 (Tcf21, marking resident fibroblasts) or myosin heavy chain 11 (Myh11, marking smooth muscle cells) promoter with a lacZ reporter. These mice were then crossed with the zsGreen reporter gene under the control of the periostin promoter. Two other non-inducible Cre were used under the control of lysozyme M (LysM, marking macrophages) or VE-cadherin (cdh5) promoters. The study revealed that less than 1% of fibroblasts were endothelial lineage traced after myocardial infarction. The majority of fibroblasts in the heart after acute myocardial infarction were derived from the Tcf21 lineage of tissue-resident fibroblasts [96]. 

In the context of coronary atherosclerosis, Cooley et al. developed a mouse model of vein graft remodeling, mimicking coronary artery bypass graft surgery, a procedure performed in patients. In this work, they show that endothelial-derived cells contribute to neointimal formation through EndMT, which proves its involvement during vein graft remodeling. They perform vein grafting in wild-type recipients using veins from both inducible and non-inducible Cre mouse models. The non-inducible model (Endotrack^YFP^) was obtained by crossing Tie2-Cre and R26RstopYFP mice, where almost 100% of EC express YFP. The inducible model (Ind.Endotrack^YFP^) was issued from crossing endSclCreER^T^ (Endothelial enhancer of the stem cell leukemia locus, EC marker) and R26RstopYFP mice. Here, 52.1% of EC were YFP positive. In these mice, endothelial-specific Cre expression is induced during adulthood by tamoxifen administration to activate the yellow fluorescence protein (YFP) gene, resulting in continuous YFP expression regardless of any change in cell phenotype. Then, a jugular vein branch from these two models was transplanted into the femoral artery of genetically matched mice. The data revealed that YFP EC-positive cells were recruited to the neointima, representing 51.7% of the total cell population originating from the EndotrackYFP and 27.8% from the inducible (Ind.EndotrackYFP) model, showing that both embryonic and adult EC is involved in neointima recruitment. They also demonstrate that ECs almost totally lost their endothelial markers (CD31, VE-cadherin) and gained α-SMA and SM22-α expression, revealing that YFP EC cells were under EndMT [97]. Another study in 2016 from the same team showed EndMT-derived fibroblasts are present in atherosclerosis using the same inducible model in ApoE-/- mice [98]. Therefore, the results compiled from these different studies show that EndMT is differently involved depending on the cardiovascular pathology. 

### 4.3. Combination of Dre-rox and Cre-loxP Models

Although inducible models are relatively efficient in labeling adult EC, the precise pattern of Cre expression is a recurrent problem since it can happen in non-targeted cells and lead to misinterpretation of the results [17]. Indeed, the precision of this system depends on the specificity of Cre expression and, thus, on the promoters being used. In 2017, He and collaborators described an interleaved dual recombinase system built to enhance the precision of conventional Cre-loxP-mediated lineage tracing [94]. This system allows the exclusion of recombination in non-targeted cells. It is based on the use of both Cre-loxP and Dre-rox systems, which both specifically excise DNA regions flanked by loxP or rox recombination sites, respectively (Figure 2C). The principle is to generate a reporter allele with the two pairs of recombination sites within the Rosa26 locus. If successful Cre-loxP recombination occurs, the first reporter will be activated (« Green » labeled cells), but not the tdTomato reporter. If successful rox recombination occurs, the tdTomato reporter will be activated (« Red » labeled cells), but not the first reporter. Due to the interleaved nature of these recombination sites, when one recombination system is activated, it inherently inhibits the ability of the second recombination system to undergo recombination. This system is also responsive to both constitutive and inducible Cre/Dre recombinases, making it extremely interesting for lineage tracing studies [94]. 

Zhang and collaborators used multiple combinations of this dual recombinase system to track EndMT in cardiac ECs and to determine whether the origins of myofibroblasts during development and cardiac fibrosis could be of endothelial origin [99]. They especially designed a triple Knock-In mouse strain (“αSMA-EndoMTracer”) in which they could trace cardiac ECs expressing α-SMA or Zeb1 markers throughout their existence. For this, they crossed mice with CreER under the control of the Cdh5 (VE-cadherin) promoter, with mice containing both αSMA-LoxP-STOP-LoxP-Dre and an interleaved sequence with the two reporters ZsGreen and tdTomato in the Rosa26 locus (Figure 2C, lower panel). After tamoxifen induction in EC, Cre recombination happens, allowing (1) Dre recombinase to be under the control of α-SMA promoter and (2) loxP recombination and activation of zsGreen promoter, labeling ECs in Green. If these cells express α-SMA during their life, Dre recombinase will be produced, allowing subsequent rox recombination in these cells, then removing zsGreen and activating tdTomato only. Therefore, in all ECs in which the α-SMA promoter has been activated even for a short time, the cells will be only labeled in red fluorescence and can be quantified (Figure 2C, lower panel). This study revealed that during development, ECs had activated α-SMA expression transiently through reversible or partial EndMT. However, during cardiac fibrosis induced by transverse aortic constriction (TAC), an acute model of pressure overload-induced cardiac hypertrophy and heart failure, the study showed that ECs did not express, even transiently, α-SMA or Zeb1 during cardiac fibrosis. 

### 4.4. Contributions and Limitations of In vivo Models

Over the last 15 years, considerable progress has been made in the field of animal models, enabling us to gain a detailed understanding of the contribution of different cell types to EndMT. The fate of endothelial cells during cardiac development and during cardiovascular pathologies can, therefore, progressively be specified, notably thanks to the use of inducible and, lastly, dual recombinase systems. The latter indeed allows us to reliably determine the specificity of Cre recombinase expression and, therefore, the origin of fibroblasts involved in fibrosis, such as endothelial cells. Again, there are a number of points that need to be addressed with the aim of making further progress in understanding their origins in order to propose relevant anti-fibrotic therapies. 

First, in recombinase systems, we cannot exclude that the gene promoter used, such as α-SMA [99], exhibits a mild level of activation, which may be insufficient to trigger recombination within a smaller subset of EC. It is also possible that some promoters, such as α-SMA or Zeb1, may not effectively label all fibroblasts, leaving some uncertainty regarding the extent of EC involvement in cardiac fibrosis [95,100]. This brings us back to the problem of the precise identification of fibroblastic markers and their specificity. Moreover, different states of fibroblasts have been identified with distinct proliferating activity and functions. Different markers are indeed expressed in 4 identified fibroblast states: resting, proliferating, activated, and resolution. Type I collagen marks all four states, whereas α-SMA is expressed in a subset of activated fibroblasts, and PDGFRα marks resting and resolution states [100]. EndMT may be induced in these possible states during cardiac fibrosis, depending on the time, the model, and the precise pathology, making the process very complex to study and analyze. This is why it is important to continue exploring the origin of fibroblasts during cardiac pathology by testing, within dual recombinase models, other relevant promoters of genes identified as specific to fibroblasts, such as type 1 collagen, PDGFRα or periostin.

Second, these studies using inducible or dual recombinase models explore the occurrence of fibrosis in the context of acute cardiac diseases, such as pressure overload induced by TAC or myocardial infarction. Results obtained from these studies clearly identify the role of EndMT in heart development, leading to the formation of resident cardiac fibroblasts but with no involvement of EndMT in cardiac fibrosis [95,96,99]. During acute myocardial injury, replacement fibrosis will occur in the form of a visible fibrotic scar triggered by ischemic cell death. Moreover, reactive fibrosis, which is a diffuse deposition and crosslinking of collagens in interstitial and perivascular areas, is observed in the remote zone of the remodeling myocardium after MI and in many non-ischemic chronic heart conditions [1]. These different types of fibrosis may not exactly involve the same mechanisms. Moreover, if we take into account the time-dependent changes in fibroblast populations in the pressure-overloaded left or right ventricle and their functional contributions to reactive and/or replacement fibrosis development, EndMT contribution may differ according to localization in the heart, pathology (especially the type of trigger in acute or chronic states), therefore favoring one or the other type of fibrosis. Indeed, in atherosclerosis, several studies show a significant involvement of EndMT-derived fibroblasts [96,97,98]. In conclusion, there is a need for further research in this area in order to assess if the involvement of EndMT could be more substantial in CVD. This will rely on studies using inducible lineage tracing models featuring relevant endothelial and mesenchymal markers and in which a chronic disease, such as diabetes mellitus or metabolic syndrome, is induced.

## 5. Conclusions

Progress in the development of various cellular and animal models over the last 15 years has provided new insights into the involvement of EndMT in cardiovascular diseases. Nevertheless, the study of this process under chronic conditions, mimicking what happens in many cardiovascular diseases, has been unexplored. Indeed, in acute pathological conditions, it would appear that EndMT has little involvement in fibrosis. On the opposite, in chronic conditions, the mechanisms may be different, with the development of interstitial fibrosis, which may involve endothelial cells-derived fibroblasts.
